# Comparative transcriptomic analysis of resistant and susceptible alfalfa cultivars (*Medicago sativa* L.) after thrips infestation

**DOI:** 10.1186/s12864-018-4495-2

**Published:** 2018-02-02

**Authors:** Xiongbing Tu, Zhongkuan Liu, Zehua Zhang

**Affiliations:** 1 State Key Laboratory for Biology of Plant Diseases and Insect Pests, Institute of Plant Protection, Chinese Academy of Agricultural Sciences, Beijing, 100193 People’s Republic of China; 2 Institute of Agro-Resourse and Environment, Hebei Academy of Agriculture and Forestry Sciences, Shijiazhuang, 050051 People’s Republic of China

**Keywords:** alfalfa cultivar resistance, thrips, transcriptomic, molecular mechanism

## Abstract

**Background:**

Plant breeding for resistance to agricultural pests is an essential element in the development of integrated crop management systems; however, the molecular and genetic mechanisms underlying resistance are poorly understood. In this pilot study, a transcriptomic analysis of a resistant (R) vs. a susceptible (S) variety of alfalfa, with (+T) or without (−T) thrips (= 4 treatments) was conducted, ‘GN-1’ (China) was defined as the resistant cultivar, and ‘WL323’ (America) was defined as the susceptible cultivar.

**Results:**

A total of 970 mRNAs were differentially expressed, of which 129 up- and 191 down-regulated genes were identified in the R + T/R-T plants, while 413 up- and 237 down-regulated genes were identified in the S + T/S-T plants. KEGG analysis mapped 33 and 80 differentially expressed genes to 11 and 14 substantially enriched pathways for GN-1 and WL323, respectively. Five shared pathways were linked to plant resistance traits, including beta-Alanine metabolism, fatty acid degradation, chloroalkane and chloroalkene degradation, flavonoid biosynthesis, and phenylalanine metabolism.

**Conclusions:**

Results indicated both thrips resistant and susceptible alfalfa cultivars can regulate gene expression in the salicylic acid (SA) and flavonoid biosynthesis pathways to induce defensive genes and protein expression (e.g. polyphenol oxidase, protease inhibitor), which enhances plant defence capacity.

**Electronic supplementary material:**

The online version of this article (10.1186/s12864-018-4495-2) contains supplementary material, which is available to authorized users.

## Background

Plant resistance breeding has long been an important component of Integrated Pest Management (IPM) [1]. Resistance provides cheap, sustainable, and environmentally safe pest control, while minimizing the use of insecticides [2]. In addition, genetic engineering now allows the insertion of exotic resistant genes into crop plants [3]. However, the molecular and genetic mechanisms underlying resistance are poorly understood. In this preliminary study, a transcriptomic study of thrips-resistant vs. thrips-susceptible alfalfa varieties was conducted to understand the molecular and genetic factors involved in plant resistance to an insect pest.

Thrips are major insect pests of alfalfa (*Medicago sativa* L.) (Fabales: Fabaceae), consuming sap from phloem tissue, removing plant nutrients and causing decreased growth, low yields, and plant death [4]. Thrips management is challenging because thrips are highly mobile, with short generation times and high reproductive rates, which allows them to quickly colonise and overwhelm plants [4]. Consequently, large quantities of insecticides are applied for their control, which adds to the cost of food production, and can cause both short and long term ecosystem damage, including non-target impacts on beneficial insects (predators, parasitoids and pollinators). Moreover, the overuse of chemicals can lead to high levels of resistance to insecticides which further complicates thrips control [5].

Mechanisms contributing to insect resistance in legumes include structural defenses, secondary metabolites and anti-nutritional compounds [6, 7]. However, the underlying genetic basis of resistance is still not well understood [8, 9]. In this study, a thrips-resistant (GN-1) and a susceptible (WL323) alfalfa cultivar was compared at the transcriptome (RNA-seq) level, each with and without thrips, to better understand the genetic and molecular mechanisms underlying plant resistance.

## Results

### Omics analyses

Two alfalfa cultivars, one resistant and the other susceptible, each with or without thrips infestation were sequenced individually, which generated ~ 56–76 million clean reads, including 8.4–11.4 G clean bases for each library (Table 1). To identify the molecular mechanism underlying these transcriptomic profiles, unigene sequences were aligned to protein databases, including NR, Swiss-Prot, KEGG and COG (e-value< 0.00001) by blastx, and to the nucleotide database NT (e-value< 0.00001) by blastn, and retrieved proteins with the highest sequence similarity to the given unigenes along with their functional annotations. Of the 143,185 unigenes, 8905 of them were annotated (Table 1).

**Table 1:** Summary of RNA-seq metrics from alfalfa cultivar transcriptomes for resistant cv. GN-1 and susceptible cv. WL323 either exposed or unexposed to thrips

Metric	GN-1 + Thrips	GN-1 - Thrips	WL323+ Thrips	WL323-Thrips
Clean reads	55,993,050	57,486,678	75,696,030	70,097,654
Clean bases (G)	8.4	8.62	11.35	10.51
Total number of Unigenes	143,185			
Annotations to Unigenes	8905			

Note: Annotations to unigenes represent the total number of unigenes with significant sequence similarity to annotated genes from NR, NT,KO, SwissProt, PFAM, GO, and KOG databases

### Differentially expressed genes (DEGs) between resistant and susceptible alfalfa cultivars

**Fig. 1:**
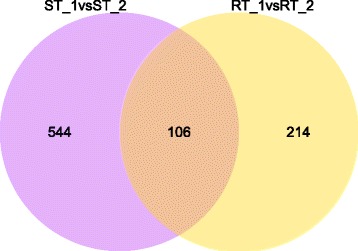
Venn diagram showing the overlap of differentially expressed genes (DEGs) related to thrips resistance of resistant alfalfa cultivar GN-1 and susceptible cultivar WL323

**Fig. 2:**
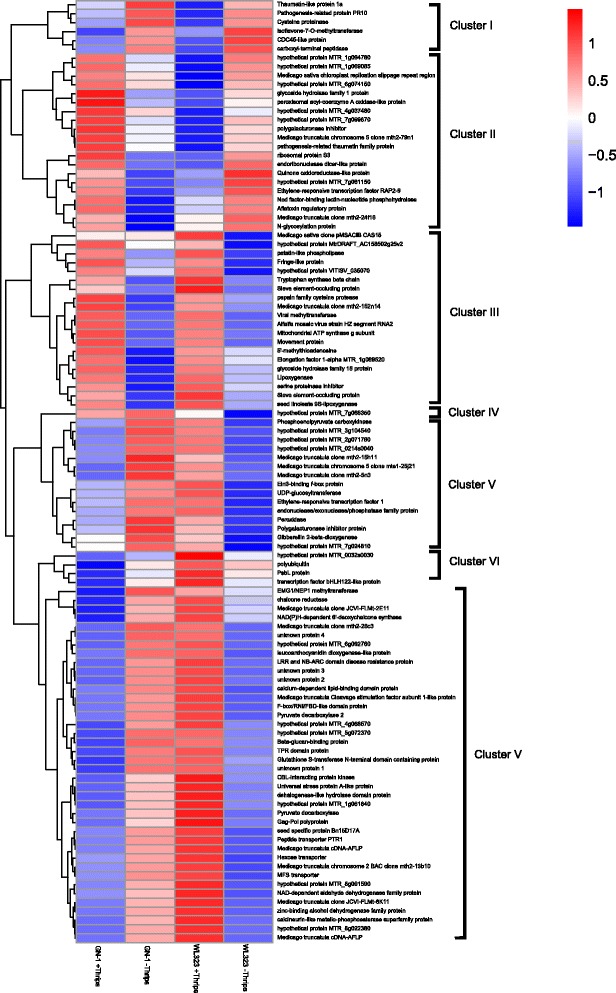
Hierarchical clustering of DEGs between thrips resistance of resistant alfalfa cultivar GN-1 and susceptible cultivar WL323. Each line in the figure represents a gene, with the columns representing thrips exposed GN-1 (R + T), unexposed GN-1 (R-T), thrips exposed WL323 (S + T) and unexposed WL323 (S-T). Red bands indicate up-regulated genes while green bands indicate down-regulated genes

Following exposure to thrips, a total of 129 and 191 up- and down-regulated transcripts, respectively, were observed in the thrips resistant cultivar GN-1 (R + T) compared to the unexposed control (R-T). For the thrips susceptible cultivar WL323, a total of 413 and 237 up- and down-regulated transcripts, respectively, were found when thrips exposed plants (S + T) were compared to the control (S-T) (FDR ≤ 0.001 and |log_2_Ratio| ≥ 1) (Table 2). Most of these transcripts were expressed within a 1- to 3-fold difference (Table 2). However, the number of DEGs found in WL323 was ~ 2 times greater than in GN-1, indicated that cellular metabolic activity in the susceptible cultivar was more active than in the resistant cultivar. Only 106 transcripts were expressed in both cultivars (Fig. 1). To identify the DEGs categories of the 106 shared transcripts, their up- or down-regulation expressions were compared, and divided into 6 clusters. **Cluster I:** Passive defence by both the resistant and susceptible cultivars, including thaumatin-like protein 1a, pathogenesis-related protein PR10, etc. For this group, all transcripts of R-T and S-T were up-regulated, while transcripts of R + T and S + T were down-regulated (Fig. 2, Additional file 1: Table S1). **Cluster II:** Active defence by the resistant cultivar and passive defence by the susceptible cultivar, including glycoside hydrolase family 1 protein, peroxisomal acyl-coenzyme A oxidase-like protein, etc. For this group, transcripts of R + T and S-T were up-regulated, while transcripts of R-T and S + T were down-regulated (Fig. 2, Additional file 1: Table S1). **Cluster III:** Active defence by the resistant and the susceptible cultivar, with or without thrips, including patatin-like phospholipase, tryptophan synthase beta chain, etc. For this cluster, all transcripts of R + T and S + T were up-regulated, while the transcripts of R-T and S-T were down-regulated (Fig. 2, Additional file 1: Table S1). **Cluster IV:** Inherent plant cultivar differences, including hypothetical protein MTR_7g068350. For this group, all transcripts of R + T and R-T were up-regulated, while transcripts of S + T and S-T were down-regulated (Fig. 2, Additional file 1: Table S1). **Cluster V:** Passive defence by the resistant cultivar and active defence by the susceptible cultivar, including CBL-interacting protein kinase, dehalogenase-like hydrolase domain protein, etc. For this cluster, transcripts of R-T and S + T were up-regulated, while transcripts of R + T and S-T were down-regulated (Fig. 2, Additional file 1: Table S1). **Cluster VI:** Active defence by susceptible cultivar, including PsbL protein, transcription factor bHLH122-like protein, etc. For this group, only transcripts of S + T were up-regulated, while transcripts of R + T, R-T and S-T were down-regulated (Fig. 2, Additional file 1: Table S1).

**Table 2:** DEGs between thrips resistant GN-1 and susceptible WL323 alfalfa cultivars. Fold change equals the read count ratio of thrips infested/uninfested

Treatment	Gene expression	Log_2_ (fold change)					
		1–2	2–3	3–4	4–5	> 5	total
GN-1 + Thrips vs GN-1 - Thrips	down	90	27	9	6	59	191
	up	56	27	13	6	27	129
	total	146	54	22	12	86	320
WL323 + Thrips vs WL323 - Thrip	down	144	48	16	9	20	237
	up	226	88	42	20	37	413
	total	370	136	58	29	57	650

### KEGG pathway analysis

To investigate the biological functions of these DEGs, 320 differentially expressed genes of the resistant (R) group were mapped to 83 pathways, while 650 differentially expressed genes of the susceptible (S) group were mapped to 165 pathways in the KEGG database. After exposure to thrips, 130 and 383 differently expressed genes from the R + T cultivar and the S + T cultivar, were assigned to reference pathways in KEGG. When both varieties were exposed to thrips, results showed only 11 and 14 biological pathways were substantially enriched (*p* < 0.05) in the R + T and the S + T groups, respectively (Table 3). Specifically, five shared pathways were highly enriched: fatty acid degradation (ko00071), phenylalanine metabolism (ko00360), beta-alanine metabolism (ko00410), chloroalkane and chloroalkene degradation (ko00625), and flavonoid biosynthesis (ko00941) (Table 3).

**Table 3:** Significantly enriched KEGG pathways in response to thrips exposure for thrips resistant cv. GN-1 and susceptible cv. WL323

Group	Num.	Term	ID	Nos. differentially expressed genes	*P*-Value
GN-1 + Thrips vs GN-1 - Thrips	1	Linoleic acid metabolism	ko00591	5	3.17E-06
	2	Isoflavonoid biosynthesis	ko00943	2	0.001014708
	3	Alanine, aspartate and glutamate metabolism	ko00250	4	0.005282324
	4	Glycolysis / Gluconeogenesis	ko00010	5	0.008999059
	5	Cutin, suberine and wax biosynthesis	ko00073	2	0.010750688
	6	beta-Alanine metabolism	ko00410	3	0.010871784
	7	Fatty acid degradation	ko00071	3	0.025783627
	8	Chloroalkane and chloroalkene degradation	ko00625	2	0.027714207
	9	Flavonoid biosynthesis	ko00941	2	0.033311014
	10	Phenylalanine metabolism	ko00360	3	0.039847515
	11	alpha-Linolenic acid metabolism	ko00592	2	0.043247811
WL323 + Thrips vs WL323 - Thrips	1	Phenylpropanoid biosynthesis	ko00940	15	1.22E-06
	2	Flavonoid biosynthesis	ko00941	6	0.000254
	3	Phenylalanine metabolism	ko00360	9	0.000487
	4	Stilbenoid, diarylheptanoid and gingerol biosynthesis	ko00945	4	0.000874
	5	Taurine and hypotaurine metabolism	ko00430	3	0.003906
	6	PPAR signaling pathway	ko03320	5	0.006629
	7	Nitrogen metabolism	ko00910	4	0.010781
	8	Arginine and proline metabolism	ko00330	7	0.013818
	9	Regulation of autophagy	ko04140	3	0.020114
	10	Plant hormone signal transduction	ko04075	8	0.036472
	11	Fatty acid degradation	ko00071	5	0.040391
	12	Chloroalkane and chloroalkene degradation	ko00625	3	0.041598
	13	beta-Alanine metabolism	ko00410	4	0.046258
	14	Valine, leucine and isoleucine degradation	ko00280	4	0.048359

To identify the functional genes potentially related to resistance in these biological pathways, 20 KEGG pathways were analyzed (Table 3). Results indicated ubiquitous genes including lipoxygenase (increased ~ 1.6-fold), tryptophan synthase beta chain (increased ~ 1.5-fold), and mitochondrial ATP synthase g subunit (increased ~ 7-fold) were up-regulated after thrips infestation. Conversely, enzymes involved in plant growth and stress response such as isoflavone-7-O-methyltransferase (decreased ~ 2.8-fold), pathogenesis-related protein PR10 (decreased ~ 2.3-fold), and Thaumatin-like protein 1a (decreased ~ 1.8-fold) were down-regulated following thrips infestation. Epidermal structure resistance genes, including sieve element-occluding proteins (increased ~ 3.1-fold), and genes related to stress tolerance, including viral methyltransferase (increased ~ 1.1-fold) and alfalfa mosaic virus strain HZ segment RNA2 (increased ~ 10-fold), were both up-regulated in the thrips resistant and susceptible plants (Additional file 1: Table S1). Genes involved in the BDG80187flavonoid biosynthesis (ko00941) pathway (and hence, in flavonoid biosynthesis and metabolism), and phenylalanine metabolism (ko00360) pathway (and thus in salicylic acid biosynthesis and metabolism) were up-regulated in both cultivars, while genes for fatty acid metabolism (ko01212) was down-regulated in both cultivars. However, when attacked by thrips, the resistant cultivar increased synthesis of linoleic acid and alpha-Linolenic acid (which can enhance cell immune reaction) as parts of the defence response to thrips feeding, but the susceptible cultivar did not show this up-regulation response (Table 3). These results showed that the immune response after thrips infestation was higher in the resistant cultivar than in the susceptible cultivar.

### PCR of DEGs

**Fig. 3:**
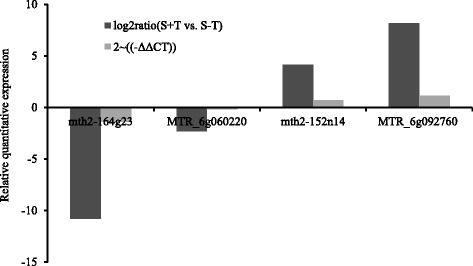
The relative quantitative expression trends of 4 DEGs by using 2^(-ΔΔCT)^ and log_2_ FPKM ratio with [(S + T)/(S-T)] methods. The 2^(-ΔΔCT)^ method to compare 4 DEGs in the S + T vs. S-T cultivars to illustrate the relative quantitative expression (Yu et al., 2007), while the log_2_ FPKM ratio showed the omics RNA sequencing results. Abbreviation: mth2-164 g23: *Medicago truncatula* chromosome 5 clone mth2-164 g23, MTR_6g060220: hypothetical protein MTR_6g060220 [*Medicago truncatula*], mth2-152n14: *Medicago truncatula* clone mth2-152n14, MTR_6g092760: hypothetical protein MTR_6g092760 [*Medicago truncatula*]

**Fig. 4:**
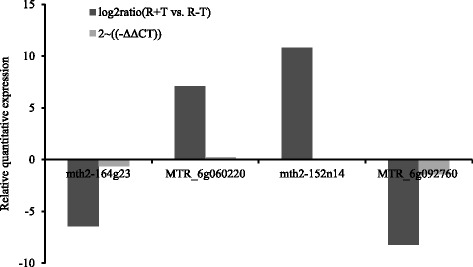
The relative quantitative expression trends of 4 DEGs by using 2^(-ΔΔCT)^ and log_2_ FPKM ratio with [(R + T)/(R-T)] methods. The 2^(-ΔΔCT)^ method to compare 4 DEGs in the R + T vs. R-T cultivars to illustrate the relative quantitative expression (Yu et al., 2007), while the log_2_ FPKM ratio showed the omics RNA sequencing results. Abbreviation: mth2-164 g23: *Medicago truncatula* chromosome 5 clone mth2-164 g23, MTR_6g060220: hypothetical protein MTR_6g060220 [*Medicago truncatula*], mth2-152n14: *Medicago truncatula* clone mth2-152n14, MTR_6g092760: hypothetical protein MTR_6g092760 [*Medicago truncatula*]

To verify the RNA sequencing results, the 4 DEGs *Medicago truncatula* chromosome 5 clone mth2-164 g23, hypothetical protein MTR_6g060220 [*Medicago truncatula*], *Medicago truncatula* clone mth2-152n14, and hypothetical protein MTR_6g092760 [*Medicago truncatula*] were selected randomly for relative quantitative analysis. In the S cultivar, the relative expression of *Medicago truncatula* chromosome 5 clone mth2-164 g23 (F = 94.53, *P* = 0.0006, Fig. 3), and hypothetical protein MTR_6g060220 (F = 0.17, *P* = 0.7029, Fig. 3) was lower in the S + T cultivar than in the S-T cultivar. For *Medicago truncatula* clone mth2-152n14 (F = 13.86, *P* = 0.0204, Fig. 3) and hypothetical protein MTR_6g092760 (F = 49.28, *P* = 0.0022, Fig. 3), the relative expression in the S + T cultivar was higher than in the S-T cultivar. In the R cultivar, the relative expression of *Medicago truncatula* chromosome 5 clone mth2-164 g23 (F = 18.73, *P* = 0.0124, Fig. 4), and hypothetical protein MTR_6g092760 (F = 876.58, *P* < 0.0001, Fig. 4) was lower in R + T cultivar than R-T cultivar. For hypothetical protein MTR_6g060220 (F = 0.97, *P* = 0.3795, Fig. 4) and *Medicago truncatula* clone mth2-152n14 (F = 0.01, *P* = 0.9145, Fig. 4), the relative expression in the R + T cultivar was higher than in the R-T cultivar. The relative quantitative expression trends of these 4 DEGs in the R + T vs. R-T and S + T vs. S-T cultivars were the same as in the omics RNA sequencing results (Figs. 3 and 4).

## Discussion

It is known that alfalfa cultivars are heterogeneous populations [10], with populations noted as resistant containing plants ranging from very susceptible to very resistant, while susceptible cultivars often have some percentage of resistant plants [11]. In a previous study, 28 cultivars were divided into resistant and susceptible cultivars based on differences in thrips numbers [10]. Two years of consecutive field observation showed biological difference between GN-1 and WL323 [10]. Hence, these two alfalfa cultivars were used as the experimental plants for omic analysis. Because the omic analysis included only a single bio-replicate in this study, we detected four differently expressed genes by PCR analysis with three bio-replicates. The PCR variation between different thrips-infested plants of each cultivar was statistically acceptable and generated rigorous datasets for analysis (Figs. 3 and 4) [12]. In this study, it is useful in finding the important differently expressed genes and proteins, and contributing to a better understanding of the genetic and molecular mechanisms underlying plant resistance.

### Plant induced resistance to thrips damage

Over the last 450 million years, land plants have evolved a vast array of anti-herbivore mechanisms, including diverse constitutive and induced defences [13]. Constitutive defences are those present before herbivore attack, and include spines, thorns, hairs, trichomes, wax, hard tissues, silicates, and many types of toxins or repellent chemicals [14], while induced defences are those that are increased by the plant following herbivore attack [13]. In this study, several induced defence genes including lipoxygenase, serine proteinase inhibitor, and seed linoleate 9S–lipoxygenase, which were up-regulated in both resistant (GN-1) and susceptible (WL323) alfalfa lines that were subject to thrips infestation, indicated that induced defences played an important role in responding to thrips attack [15–17]. Furthermore, five shared pathways were found in both the resistant alfalfa variety (GN-1) and the susceptible variety (WL323), including fatty acid degradation (ko00071;related to energy metabolism), chloroalkane and chloroalkene degradation (ko00625; related to xenobiotics biodegradation and metabolism), beta-alanine metabolism (ko00410) and phenylalanine metabolism (ko00360; both related to salicylic acid synthesis [18]), and flavonoid biosynthesis (ko00941; flavonoids represent an important secondary defence metabolic pathway associated with plant defences [19, 20]). All indicate that both thrips resistant and susceptible alfalfa cultivars can regulate gene expression in the salicylic acid and flavonoid biosynthesis pathways to induce expression of defensive genes and proteins expression (e.g. polyphenol oxidase, protease inhibitor), which is known to enhance defence capacity [21–24].

### Function cluster of differentially expressed genes

Differentially expressed genes (DEGs) and biological pathways analysis showed that 106 transcripts were differently expressed and may serve various roles in alfalfa cultivar resistance (Fig. 2). In this study, all DEGs were grouped into six clusters. Five of the clusters were associated with plant responses to thrips attack including both active and passive defences in both the resistant and susceptible cultivars. The sixth cluster was related to inherent cultivar differences. This result is crucial for future related work, as it helps to identify key genes related to thrips resistance in an important forage crop, and offers not only the opportunity to enhance breeding but also provides the opportunity to incorporate key resistance genes into cultivars that have valuable agronomic characteristics (e.g. high yield, tolerant to grazing) but are otherwise constrained by poor performance due to low resistance to thrips [25].

## Conclusions

In this study, it demonstrated that the number of DEGs found in the susceptible cultivar was about twice as high as the number of DEGs in the resistant cultivar, indicating that cellular metabolic activity in the susceptible cultivar was more active than in the resistant cultivar. However, the immune response after thrips infestation was higher in the resistant cultivar than in the susceptible cultivar, as the resistant cultivar can regulate linoleic acid and alpha-Linolenic acid synthesis, and induce an immune response to thrips feeding. Most importantly, genes associated with the flavonoid biosynthesis pathway (ko00941), and of the beta-alanine metabolism (ko00410) and phenylalanine metabolism (ko00360) pathways (both related to SA synthesis), were up-regulated in both cultivars, this indicates that the expression of flavonoids and SA plays an important role in regulating plant response to thrips feeding.

## Methods

### Sample preparation

Two alfalfa cultivars were selected based on the results of field experiments evaluating plant resistance to thrips [26]. Gongnong 1 (GN-1, China) is classified as an thrips-resistant, and WL323 (America) as an thrips-susceptible cultivar [26]. Seeds from each cultivar were planted into pots containing field collected soil, after which the pots were placed outside under ambient conditions to germinate and grow. Plants were watered 2 to 3 times per week as required. To exclude thrips, the plants were protected within a cage covered in a fine mesh cloth. After ~ 35 d, when the plants had reached 50% budding stage, one of the plants from each cultivar was selected and 30 *Odontothrips loti* (Haliday), placed onto the leaves and left for a further 72 h under ambient conditions. The control treatments had no thrips and were maintained under the same conditions. There were four treatments: GN-1 plus (R + T) or minus (R-T) thrips and WL323 plus (S + T) or minus (S-T) thrips with only one replicate per treatment. Thrips populations on WL323 were well established at the end of the three days, with ~ 23 thrips, compared to GN-1 where only ~ 7 thrips were present. At the conclusion of the 72-h thrips exposure, the top 3–4 new leaves were removed from each treatment, cleaned of any thrips, and placed in separate plastic bags. Samples were frozen within 20 min at -80 °C for omics analysis.

### RNA extraction, library construction and sequencing

Alfalfa cultivars were used from each of the four treatments, RNA extraction, library construction and sequencing were carried out as described previously [27].

### Sequence reads, mapping, assembly and annotation

All the downstream analyses were based on clean data with high quality, and the methods for sequence reads and mapping could be referenced as Hao et al. [27]. The left.fq and right.fq were used for the transcriptome assembly [27], and seven databases including Nt (NCBI non-redundant nucleotide sequences), Nr (NCBI non-redundant Protein sequences), Pfam (Protein family), KO (KEGG Ortholog database) and GO (Gene Ontology), KOG/COG (Clusters of Orthologous Groups of proteins), Swiss-Prot (a manually annotated and reviewed protein sequence database) were used for gene function annotation [27]. Data for each sequenced library was analyzed using BLAST with a cutoff E-value of 10^− 5^.

### Differential expression gene analysis

Prior to differential gene expression analysis, for each sequenced library, the read counts were adjusted using the edge R program package through one scaling normalized factor. Differential expression gene analysis of two samples was performed using the DEGseq R package [28]. *P* value was adjusted using q value [29]. q value < 0.005 & |log_2_(fold change) | > 1 was set as the threshold for significantly differential expression.

### KEGG enrichment analysis of differentially expressed transcripts

KEGG is a database resource for understanding high-level functions and utilities of the biological system [30], whether the cell, the organism or the ecosystem and is derived from molecular-level information, especially large-scale molecular datasets generated by genome sequencing and other high-throughput experimental technologies (http://www.genome.jp/kegg/). KOBAS software was used to test the statistical enrichment of differential expression genes in KEGG pathways [31].

### PCR of DEGs

Sequences of 4 DEGs including *Medicago truncatula* chromosome 5 clone mth2-164 g23 (c67284_g1), hypothetical protein MTR_6g060220 (c90193_g2), *Medicago truncatula* clone mth2-152n14 (c775_g1), and hypothetical protein MTR_6g092760 (c31797_g1) were obtained randomly from the transcriptome datasets. PCR primers were designed for each of these 4 genes and were used to determine if the genes were expressed in the EST pools of R + A vs. R-A cultivars. PCR was performed as following conditions: 95 °C for 3 min; 40 cycles of 94 °C for 20 s, 55 °C for 20 s and 72 °C for 20 s; final at 72 °C for 5 min, three replicates per treatment [32]. The expression ratios calculated by using 2^(-ΔΔCT)^ method [33].

## Additional file

Additional file 1: Table S1. All differentially expressed genes (DEGs) description between thrip resistant GN-1 and susceptible WL323 alfalfa cultivars. (XLSX 20 kb)

## Abbreviations

ATP: adenosine triphosphate
DEGs: Differentially expressed genes
FDR: False Discovery Rate
GN-1: Gongnong 1 (alfalfa variety)
GO: Gene Ontology
IPM: Integrated Pest Management
KEGG: Kyoto Encyclopedia of Genes and Genomes
KO: KEGG Ortholog database
KOG/COG: Clusters of Orthologous Groups of proteins
NR: NCBI non-redundant protein sequences
NT: NCBI nucleotide sequences
Pfam: Protein family
R: Resistant
S: Susceptible
SA: Salicylic acid
Swiss-Prot: A manually annotated and reviewed protein sequence database
T: Thrips

## References

[1] Cloyd RA (2016). Western flower Thrips (Thysanoptera: Thripidae) and insecticide resistance: an overview and strategies to mitigate insecticide resistance development. J Entomol Sci.

[2] Kozjak P, Meglič V, Mishra R (2012). Mutagenesis in plant breeding for disease and Pest resistance, chapter 10, pp.195-220. Biochemistry.

[3] Lu Y, Wu K, Jiang Y, Guo Y, Desneux N (2012). Widespread adoption of Bt cotton and insecticide decrease promotes biocontrol services. Nature.

[4] Zhang R, Yang F, Xian CZ, Ma JH, Zhang SH (2005). A study on the yield loss and economic threshold of alfalfa damaged by thrips, *Odentothrips lati*. Plant Prot.

[5] Zhang R, Ma JH, Yang F, Xian CZ, Zhang SH. Field tests of several insecticides to control alfalfa thrips. Pratacultural sci 2004; 21 (1): 20-21. (in Chinese).

[6] Edwards O, Singh KB (2006). Resistance to insect pests: what do legumes have to offer?. Euphytica.

[7] Leiss KA, Cristofori G, van Steenis R, Verpoorte R, PGL K (2013). An eco-metabolomic study of host plant resistance to western flower thrips in cultivated, biofortified and wild carrots. Phytochemistry.

[8] Zhang SY, Kono S, Murai T, Miyata T (2008). Mechanisms of resistance to spinosad in the western flower thrip, *Frankliniella occidentalis* (Pergande) (Thysanoptera: Thripidae). Insect Sci.

[9] Cifuentes D, Chynoweth R, Guillén J, De La Rúa P, Bielza P (2012). Novel Cytochrome P450 genes, CYP6EB1 and CYP6EC1, are over-expressed in Acrinathrin-resistant *Frankliniella occidentalis* (Thysanoptera: Thripidae). J Econ Entomol.

[10] Bagavathiannan MV, Julier B, Barre P, Gulden RH, Van Acker RC (2010). Genetic diversity of feral alfalfa (Mediccago sativa L.) populations occurring in Manitoba, Canada and comparison with alfalfa cultivars: an analysis using SSR markers and phenotypic traits. Euphytica.

[11] Buggle P, Gutierrez AP (1995). Use of life table to assess host plant resistance in alfalfa to *Therioaphis trifolii* f. *maculata* (Homoptera: Aphididae): hypothesis for maintenance of resistance. Environ Entomol.

[12] Liu D, Xu LS, Vandemark G, Chen WD (2016). Comparative Transcriptome analysis between the fungal plant pathogens *Sclerotinia sclerotiorum* and *S. trifoliorum* using RNA sequencing. J Hered.

[13] Whitman DW, Ananthakrishnan TN (2009). Phenotypic plasticity of insects: mechanisms and consequences.

[14] Paiva NL (2000). An introduction to the biosynthesis of chemicals used in plant-microbe communication. J Plant Growth Regul.

[15] Maleck K, Dietrich RA (1999). Defense on multiple fronts: how do plants cope with diverse enemies?. Trends Plant Sci.

[16] Stotz HU, Kroymann J, Mitchell-Olds T (1999). Plant-insect interactions. Curr Opin Plant Biol.

[17] Walling LL (2000). The myriad plant responses to herbivores. J Plant Growth Regul.

[18] Leon-Reyes A, Van der Does D, De Lange ES (2010). Salicylate-mediated suppression of jasmonate-responsive gene expression in Arabidopsis is targeted downstream of the jasmonate biosynthesis pathway. Planta.

[19] MSJ S (2003). Flavonoid-insect interactions: recent advances in our knowledge. Phytochemistry.

[20] JDG J, Dangl JL (2006). The plant immune system. Nature.

[21] Prince DC, Drurey C, Zipfel C, Hogenhout SA (2014). The leucine-rich repeat receptor-like kinase brassinosteroid insensitive 1- associated kinase 1 and the cytochrome P450 phytoalexin deficient 3 contribute to innate immunity to aphids in *Arabidopsis*. Plant Physiol.

[22] Tzin V, Fernandez-Pozo N, Richter A, Schmelz EA, Schoettner M, Schäfer M, Ahern KR, Meihls LN, Kaur H, Huffaker A, Mori N, Degenhardt J, Mueller LA, Jander G (2015). Dynamic maize responses to aphid feeding are revealed by a time series of Transcriptomic and Metabolomic assays. Plant Physiol.

[23] Kloth KJ, Wiegers GL, Busscher-Lange J, van Haarst JC, Kruijer W, Bouwmeester HJ, Dicke M, Jongsma MA (2016). AtWRKY22 promotes susceptibility to aphids and modulates salicylic acid and jasmonic acid signalling. J Exp Bot.

[24] Züst T, Agrawal AA (2016). Mechanisms and evolution of plant resistance to aphids. Nat Plants.

[25] Smith CM, Clement SL (2012). Molecular bases of plant resistance to arthropods. Annu Rev Entomol.

[26] Tu XB, Fab YL, Ji MS, Liu ZK, Xie N, Liu ZY, Zhang ZH (2016). Improving a method for evaluating alfalfa cultivar resistance to thrips. J Integr Agri.

[27] Hao K, Wang F, Nong XQ, McNeill MR, Liu SF, Wang GJ, Cao GC, Zhang ZH (2017). Response of peanut Arachis Hypogaea roots to the presence of beneficial and pathogenic fungi by transcriptome analysis. Sci Rep.

[28] Anders S, Huber W (2010). Differential expression analysis for sequence count data. Genome Biol.

[29] Storey JD, Tibshirani R (2003). Statistical significance for genome wide studies. PNAS.

[30] Kanehisa M, Araki M, Goto S (2008). KEGG for linking genomes to life and the environment. Nucleic Acids Res.

[31] Mao X, Cai T, Olyarchuk JG, Wei L (2005). Automated genome annotation and pathway identification using the KEGG Orthology (KO) as a controlled vocabulary. Bioinformatics.

[32] Hegedus DD, Rimmer SR (2005). Sclerotinia sclerotiorum: when “to be or not to be” a pathogen?. FEMS Microbiol Lett.

[33] Yu L, Liu JP, Zhuang ZX, Yang LQ, Zhang RL, Ye XM, Cheng JQ (2007). Quantitative analysis of real-time PCR expression production by REST and 2~((-ΔΔCT)). J Trop Med.

